# Targeting protein arginine methyltransferase 5 inhibits human hepatocellular carcinoma growth via the downregulation of beta-catenin

**DOI:** 10.1186/s12967-015-0721-8

**Published:** 2015-11-05

**Authors:** Baolai Zhang, Shuhong Dong, Zhongxin Li, Li Lu, Su Zhang, Xue Chen, Xiaobo Cen, Yongjie Wu

**Affiliations:** Department of Pharmacology, School of Basic Medical Sciences, Lanzhou University; Key Lab of Preclinical Study for New Drugs of Gansu Province, No 199, Dongang West Road, Lanzhou, 730000 Gansu China; Gansu Provincial Second People’s Hospital, Lanzhou, 730000 China; State Key Laboratory of Biotherapy and Cancer Center, West China Hospital, Sichuan University, and Collaborative Innovation Center for Biotherapy, Chengdu, 610041 China

**Keywords:** PRMT5, HCC, β-Catenin, AMI-1, H4R3, H3R8 methylation

## Abstract

**Background:**

Protein arginine methyltransferase 5 (PRMT5), a type II PRMT, is highly expressed in some tumors, but its role in hepatocellular carcinoma (HCC) is still unknown.

**Methods:**

PRMT5 level in HCC specimens was determined by immunohistochemical staining and the association with clinicopathologic features was evaluated. PRMT5 was inhibited by AMI-1 (a small molecule inhibitor of PRMTs) or small interference RNA (siRNA). The proliferation of HCC cells was tested by Cell Counting Kit-8, cell migration was evaluated by Transwell assay and cell cycle and apoptosis were analyzed by flow cytometry. The effect of AMI-1 on HCC in vivo was examined by mouse xenograft model.

**Results:**

PRMT5 expression was markedly upregulated in HCC tissues, and correlated inversely with overall patient survival. Knockdown of PRMT5 significantly reduced the proliferation of HCC cells, but did not affect the growth of normal liver cells. Furthermore, β-catenin was identified as a target of PRMT5. Silencing PRMT5 significantly down-regulated the expression of β-catenin and the downstream effector Cyclin D1 in HCC cells. AMI-1 strongly inhibited HCC growth in vivo, increased the ratio of Bax/Bcl-2, and led to apoptosis and loss of migratory activity in several HCC cells. Meanwhile, AMI-1 decreased the expression levels of symmetric dimethylation of H4 (H4R3me2s), a histone mark of PRMT5.

**Conclusions:**

PRMT5 plays an important role in HCC. PRMT5 may be a promising target for HCC therapy.

**Electronic supplementary material:**

The online version of this article (doi:10.1186/s12967-015-0721-8) contains supplementary material, which is available to authorized users.

## Background

Hepatocellular carcinoma (HCC) is the fifth leading cancer and the third most common cause for cancer death worldwide [1–3]. The overall survival of patients with HCC is less than 10 %. Surgical resection and liver transplantation is the main therapeutic strategy for HCC if patients are diagnosed at an early stage [4]. However, a majority of patients with HCC present with advanced disease and treatment options are limited [5]. Sorafenib has been recognized as the most effective targeted therapeutic agent for advanced HCC. However, compared with placebo groups, sorafenib only increased survival from 7.9 to 10.7 months [6]. Therefore, it is urgent to develop more effective therapeutic strategies and agents to treat HCC.

Protein arginine methyltransferase 5 (PRMT5), a type II arginine methyltransferase, is localized in both cytoplasm and the nucleus of mammalian cells [7, 8]. PRMT5-driven methylation of arginine residues leads to symmetric dimethylation of histone H3 (H3R8me2s) and H4 (H4R3me2s), which in turn alter chromatin structure to regulate gene expression [9, 10]. PRMT5 has been found to be overexpressed in multiple tumor types, including leukemia, lymphoma, lung cancer, colorectal cancer and breast cancer [11–15]. However, the role of PRMT5 in hepatocarcinogenesis has not been established.

In this study we aimed to investigate the role of PRMT5 in hepatocarcinogenesis by using in vitro and in vivo models. We showed that PRMT5 protein is overexpressed in HCC tumor tissues, and its elevated level is associated with worse prognosis in patients with HCC. In addition, we found that specific deletion of PRMT5 by small interference RNA (siRNA) or the inhibition of its activity by arginine methyltransferase inhibitor 1 (AMI-1) significantly decreases HCC development. Furthermore, we demonstrate that the deletion of PRMT5 significantly down-regulates the expression of β-catenin and its downstream effector Cyclin D1 in HCC cells.

## Methods

### Human tissue samples and cell lines

Fifty-four pairs of HCC tissues and corresponding normal adjacent tissues (NATs) were purchased from the National Engineering Center for Biochips (Shanghai, China). All human materials were obtained with informed consent, and this study was approved by Ethics Committee of Lanzhou University. Four human HCC cell lines (HepG2, Bel-7404, Bel-7402 and SMMC-7721), one rat hepatoma cell line CBRH-7919 and one normal liver cell line HL-7702 were purchased from American Type Culture Collection (Manassas, VA, USA), and cultured under conditions recommended.

### Immunohistochemical staining

Tissue arrays were constructed using 54 pairs of HCC tissues and corresponding NATs. Immunohistochemical staining was performed on 4 μm sections of paraffin-embedded human HCC tissues and matched NATs to determine the expression level of PRMT5 protein. Briefly, the slides were incubated with PRMT5 antibody at 4 °C overnight. After extensive washing, the sections were incubated with secondary antibody, followed by staining with DAB (ZSGB-BIO, Beijing, China). The slides were then washed and counterstained with hematoxylin. A semiquantitative scoring method according to the overall staining of the cells was applied for immunohistochemistry (IHC) [16, 17].

### RNA interference

Three different siRNA each were synthesized against human PRMT5 by Ribobio (Guangzhou, China), and the most effective one (target sequence: CCGCTATTGCACCTTGGAA) was selected for the subsequent experiments with test siRNA. Cells were transfected with PRMT5 siRNA (si-PRMT5, final concentration of 50 nM) or scramble siRNA (Negative control siRNA, si-NC) using Lipofectamine 2000 (Invitrogen, Carlsbad, CA, USA) according to manufacturer’s protocol.

### Cell proliferation assay and colony formation assay

Cells were seeded in flat-bottom 96-well plates (2.5 × 10^3^ cells per well) in triplicate. After 24 h, cells were treated with si-NC, si-PRMT5 or AMI-1. Then cell proliferation was assessed using the Cell Counting Kit-8 (Dojindo, Tokyo, Japan) according to the manufacturer’s protocols. The efficacy of the treatment was expressed as best tumor growth inhibition [18, 19]. The *T*/*C* value was calculated as follows: %*T*/*C* = (median tumor weight of treated tumors/median tumor weight of control tumors) × 100. For the colony formation assay, HepG2 and HL-7702 cells were placed in 60 mm dishes at 200 cells per dish and maintained in the absence or presence of si-PRMT5 for 16 days. Cells were fixed with fixative (7 parts methanol: 1 part glacial acetic acid) for 15 min and then stained with crystal violet (0.2 g/L) for 30 min.

### Cell cycle analysis and apoptosis assay

Cell cycle and apoptosis were analyzed by flow cytometry as described previously [20, 21]. Briefly, cells were seeded at 1 × 10^5^ cells/well in 6-well plates and transfected with si-NC or si-PRMT5. After 72 h, cells were harvested using 0.25 % trypsin without EDTA and fixed in 75 % ethyl alcohol at −20 °C overnight. The next day, cells were exposed to 40 mg/ml propidium iodide and 100 mg/mL ribonuclease A in PBS for 30 min at room temperature in the dark. Cell DNA content was then analyzed using FACS. For apoptosis assay, the treated cells were harvested using 0.25 % trypsin, washed twice and resuspended in Binding Buffer at concentration of 1 × 10^6^ cells/mL. 500 µL of cells were incubated with 5 µL of Annexin V-FITC and 5 µL of Propidium Iodide for 5 min at room temperature in the dark. The samples were then evaluated by FACS (Navios, Beckman Coulter, CA, USA).

### Western blot analysis

Cell lysates were separated in 12 % SDS–polyacrylamide gel electrophoresis, and then transferred to PVDF membrane (Bio-Rad). After being blocked with 5 % non-fat milk for 1 h, the membranes were incubated with the following primary antibodies at 4 °C overnight: PRMT5 (1:400; Santa Cruz Biotech, Santa Cruz, CA, USA), PRMT7 (1:500; Santa Cruz Biotech), Cyclin D1 (1:500; Santa Cruz Biotech), CTNNB1 (β-catenin, 1:1000; ABclonal, MA, USA), H4R3me2s (1:2000; Abcam, MA, USA), H3R8me2s (1:250; Novus, CO, USA), Bax (1:1000; Cell Signaling, MA, USA), Bcl-2 (1:1000; Cell Signaling), PRMT7 (1:1000; Cell Signaling) or β-actin (1:2000; Cell Signaling), and then incubated with secondary antibodies conjugated to horseradish peroxidase (ZSGB-BIO, Beijing, China). Immuno-reactivity was visualized using ECL Western blotting detection reagents and then analyzed by densitometry.

### Migration assay

Cell migration assay was performed in 24-well transwell chambers, 8 µm pore size Transwell plate (Corning Inc.; NY, USA) [22, 23]. In brief, the cells resuspended in 100 µL serum-free medium (4 × 10^4^ cells per well) were placed in upper chamber of insert in triplicate and medium with 600 µL 10 % FBS was used as chemo-attractant in lower chamber. AMI-1 or vehicle control was added to inner chamber. After 24 h, the cells remaining on the top surface of the membrane were removed with a cotton swab. The cells on the bottom surface of the membrane were fixed in methanol and stained with 0.2 % crystal violet.

### Human HCC xenograft model

Female athymic BALB/c nude mice were purchased from Shanghai SLAC Laboratory Animal Co. Ltd (Shanghai, China) and maintained under specific pathogen-free room with a 12-h on/off light cycle, and fed autoclaved chow and water. Mice were manipulated and housed according to protocols approved by the Institutional Animal Care and Treatment Committee of Lanzhou University. 5 × 10^6^ HepG2 cells were inoculated subcutaneously (s.c) in the right flank of 5–6-week old healthy BALB/c nude mice. When tumors reached an average volume of about 70 mm^3^, the mice bearing too large or too small tumors were eliminated and the left were randomly divided into two treatment groups (8 animals per group): AMI-1 (0.5 mg in 100 µL of 0.9 % NaCl) or 100 µL of 0.9 % NaCl were administered intratumorly (i.t) twice a week. Control animals and animals treated with AMI-1 were sacrificed after 36 days and tumor were excised and weighted. Tumor volumes were calculated according to the following formula: (*length* × *width*^2^) × 0.52.

### Statistical analysis

Unless otherwise stated, data were presented as the mean ± SD and subjected to student’s *t* test. Differences were considered statistically significant as *P < 0.05, **P < 0.01 or ***P < 0.001.

## Results

### PRMT5 is overexpressed in primary HCC tumors

**Fig. 1:**
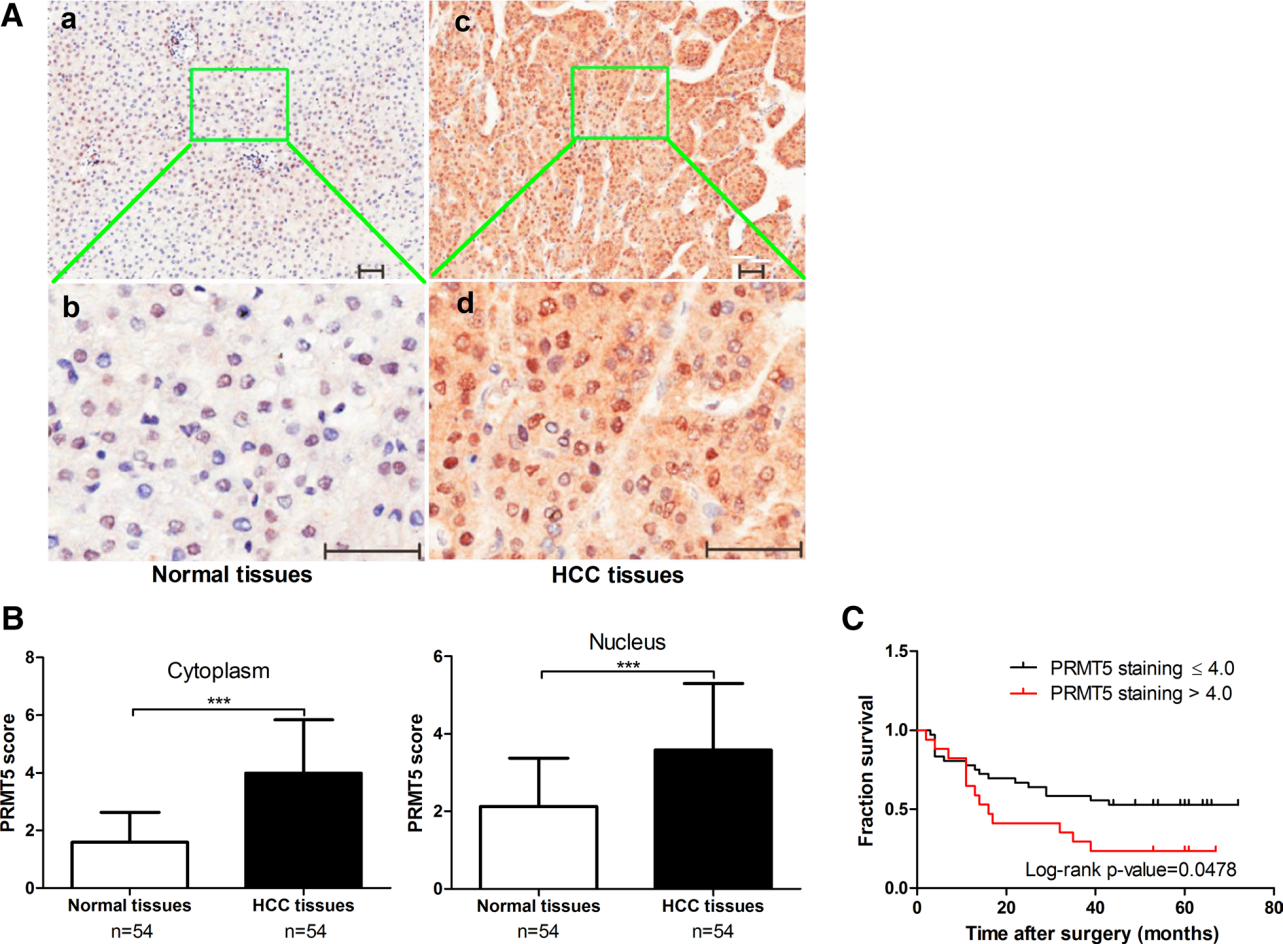
Upregulation of PRMT5 in HCC tissues and its association with poor survival. **A** Immunohistochemical staining of PRMT5 in HCC tissues and corresponding NATs. Positive cells were stained brown. Magnification, (*a*,* c*) ×50; (*b*,* d*) ×100; *scale bar* 50 μm. **B** PRMT5 scores based on the nuclear or cytoplasmic levels of expression in 54 HCC patients, compared with matched normal tissues. **C** Association between PRMT5 expression and the survival of HCC patients

To evaluate the clinical significance of PRMT5 in HCC development, we analyzed PRMT5 level and its distribution in HCC tissues by immunohistochemical (IHC) staining. The array included the cancer tissue samples and corresponding normal adjacent tissues (NATs) from 54 HCC patients, which consisted of 48 males and 6 females, with a medium age of 53 years (range 38–72 years). Among the patients 31 (57.4 %) died of tumor related to causes, and 23 (42.6 %) were still alive as of the last follow-up (Table 1). PRMT5 levels in the tissues array specimens were assessed based on the staining density scores. We found that in cancer tissues, 31 cases (57.4 %) exhibited strong immunopositivity, 18 cases (33.3 %) exhibited moderate immunopositivity, and 5 cases (9.3 %) exhibited no or weak immunopositivity. In contrast, the majority of normal tissues (51.9 %) exhibited no or weak PRMT5 expression (Table 2). Statistical analysis revealed that PRMT5 level significantly increased in cancer tissues compared with the corresponding NATs (Fig. 1A, B). In addition, cytoplasmic PRMT5 level, but not nuclear PRMT5 level, had a trend to be negatively correlated with the survival rate of the patients with HCC (Fig. 1C). As negative control, PRMT5 antibody could not detect positive staining (Additional file 1: Figure 1). Notably, no significant association was found between PRMT5 expression in HCC and tumor size or TNM stage (*P* > 0.05, Table 1). Univariate Cox regression analyses showed that PRMT5 expression in the cytoplasm was significantly correlated with overall survival (Table 3). Furthermore, multivariate Cox regression analysis confirmed that cytoplasmic PRMT5 expression could serve as independent predictor of the overall survival of patients with HCC (Table 3). In addition, we performed Western blot analysis to determine of PRMT5 protein level in several HCC cell lines. As shown in Additional file 1: Figure 2, PRMT5 was expressed in normal liver HL-7702 cells and highly expressed in Bel-7704, HepG2 and CBRH-7919 HCC cell lines. Taken together, these results indicate that PRMT5 overexpressed is tightly linked to HCC proliferation and development.

**Table 1:** Clinical and pathological characteristics of patients with HCC (n = 54)

Characteristics	n	%	PRMT5 score	P value
Gender				
Male	48	88.9	3.8542	0.369
Female	6	11.1	3.3333	
Age (years)				
≤50	17	31.5	3.2059	0.034
>50	37	68.5	4.0135	
Tumor size (cm)				
≤5	27	50	3.8611	0.573
>5	27	50	3.6574	
TNM stage				
I	6	11.1	4.0833	0.259
II	37	68.5	3.6757	
III	11	20.4	8.8636

**Table 2:** Summary of PRMT5 scoring distribution

Composite score	Normal liver tissue (n = 54)	HCC tissue (n = 54)
<2 (weak immunopositivity)	28 (51.9 %)	5 (9.3 %)
2–4 (moderate immunopositivity)	22 (40.7 %)	18 (33.3 %)
≥4 (strong immunopositivity)	4 (7.4 %)	31 (57.4 %)
P values		<0.0001

*P* value calculated by *χ* ^2^ test for normal liver tissue versus HCC tissue

**Table 3:** Univariate and multivariate analyses of the survival of HCC patients

Covariate	n	Univariate analysis			Multivariate analysis		
		HR	95 % CI	*P* value	HR	95 % CI	*P* value
Gender				0.710			0.758
Male	48	1.000			1.000		
Female	6	1.254	0.381–4.126		1.232	0.326–4.654	
Age (years)				0.812			0.371
≤50	17	1.000			1.000		
>50	37	1.099	0.506–2.387		0.671	0.280–1.609	
Tumor size (cm)				0.037			0.062
≤5	27	1.000			1.000		
>5	27	2.170	1.046–4.504		2.148	0.964	4.785
TNM stage				0.105			0.329
I	6	1.000			1.000		
II	37	1.087	0.323–3.659				
III	11	2.395	0.633–9.058		1.459	0.683–3.116	
Cytoplasmic PRMT5				0.033			0.029
Low	36	1.000			1.000		
High	18	2.175	1.067–4.435		2.411	1.094–5.313	
Nuclear PRMT5				0.963			0.691
Low	40	1.000			1.000		
High	14	1.020	0.439–2.369		1.228	0.446–3.380	

The Cox proportional hazards regression model was used for univariate and multivariate analyses to study the effects of the clinicopathological variables and PRMT5 expression on survival

*HR* hazard ratio, *CI* confidence interval

### Knockdown of PRMT5 suppresses HCC cell proliferation in vitro

**Fig. 2:**
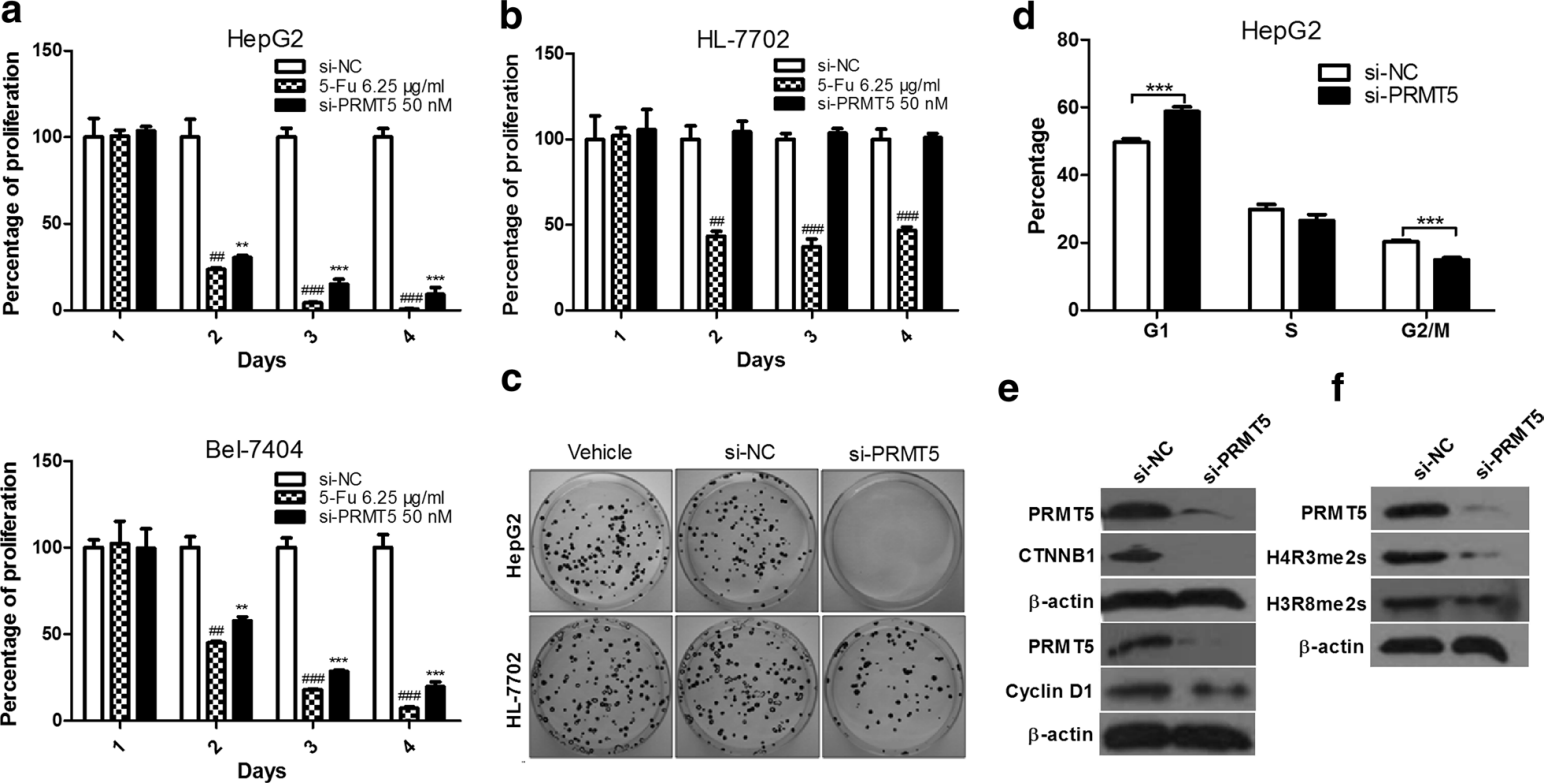
Silencing PRMT5 decreases human HCC cell growth in vitro. **a** HepG2 and Bel-7404 cells were transfected with PRMT5 siRNA (si-PRMT5) or scramble negative control siRNA (si-NC) and cell proliferation was analyzed. **b** Normal liver HL-7702 cells were transfected with PRMT5 siRNA (si-PRMT5) or scramble negative control siRNA (si-NC) and cell proliferation was analyzed. **c** The effect of si-PRMT5 on colony formation of HepG2 and HL-7702 cells. Representative results of colony formation of vehicle (*left*), si-NC (*middle*), and si-PRMT5 (*right*) HepG2 and HL-7702 cells. 5-fluorouracil (5-Fu) is used as a positive control. **d** Knockdown of PRMT5 led to G1 arrest. HepG2 cells were transfected with si-NC or si-PRMT5 and then subjected to cell cycle analysis. **e** and **f** Western blot analysis of whole-cell lysates derived from HepG2 cells transfected with si-NC or si-PRMT5 using antibodies against PRMT5, β-catenin, Cyclin D1, H4R3me2s or H3R8me2s

To determine the role of PRMT5 in HCC cell proliferation, we employed siRNA against human PRMT5 to knockdown PRMT5 in two human HCC cells (HepG2 and Bel-7404) and one normal liver cell (HL-7702), and then cell proliferation was measured by CCK-8 assay. As shown in Fig. 2a–c, silencing PRMT5 significantly decreased proliferation and colony formation of HCC cells, but not normal hepatocyte HL-7702. In addition, because PRMT5 and PRMT7 have been shown to possess type II methyltransferase activity [24, 25], we tested the specificity of si-PRMT5 in HCC cell lines. The results showed that si-PRMT5 did not affect the protein levels of PRMT7 (data not shown).

### Knockdown of PRMT5 induces HCC cell cycle arrest

To explore the mechanism by which PRMT5 knockdown inhibits HCC cell proliferation, we performed cell cycle analysis. As shown in Fig. 2d, PRMT5 knockdown led to an increase of cell population at the G1 phase, with a corresponding decrease in S and G2/M phase, compared with si-NC, indicating that PRMT5 may be required for the G1-to-S phase transition.

To understand underlying mechanism of cell cycle arrest, the levels of several cell proliferation/cycle-related proteins in si-NC and PRMT5-knockdown HCC cells were analyzed by Western blot analysis. As shown in Fig. 2e, knockdown of PRMT5 significantly decreased the expression of β-catenin and Cyclin D1 in HCC cells. These results indicate that PRMT5 promotes cell cycle progression by regulating the expression of cell cycle-related proteins such as β-catenin and Cyclin D1.

Because PRMT5-driven methylation of arginine residues leads to H4R3me2s and H3R8me2s, we then measured H4R3me2s and H3R8me2s in HCC cells treated with si-PRMT5. We found that the levels of H4R3me2s and H3R8me2s were significantly decreased compared with si-NC (Fig. 2f).

### AMI-1 inhibits HCC cell proliferation in vitro and in vivo

**Fig. 3:**
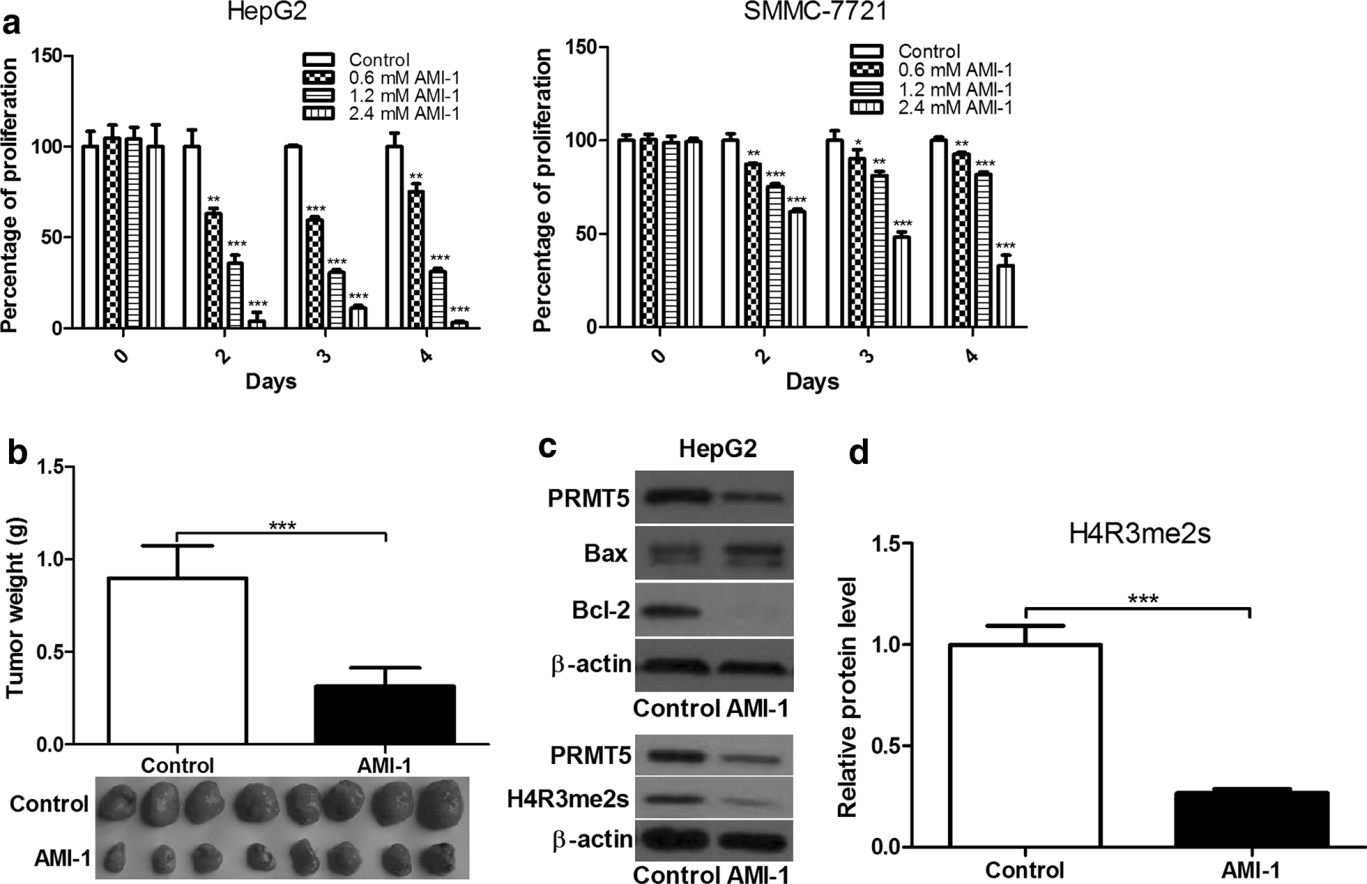
AMI-1 inhibits HCC cell growth in vitro and in vivo. **a** The effect of AMI-1 on the proliferation of human HCC cell lines. **b** The effect of AMI-1 on tumor formation in a nude mouse xenograft model. **c** The expression of Bax, Bcl-2 and H4R3me2s in HepG2 cells treated with AMI-1 for 72 h. **d** Densitometric analysis of band intensities. β-actin was loading control. Control refers to vehicle-treated group

AMI-1 has been applied to inhibit type I PRMT (PRMT1, 3, 4, and 6) activity in vitro [26]. Interestingly, we found that AMI-1 also inhibited 84.2 % of type II PRMT5 activity at the tested concentration (nearly 50 µM) [27]. Therefore, we examined the in vitro and in vivo efficacy of AMI-1 on HCC using human HCC cell lines and xenograft mouse models. The concentrations of AMI-1 used for in vitro and in vivo experiments and for enzymatic assay are different, based on our preliminary experiments and previous literatures [27–30]. As shown in Fig. 3a, AMI-1 elicited a significant inhibition on HCC cell growth. In animal tumor models, the tumors were injected with AMI-1 intratumorly (i.t.), because the drug via systemic delivery is easily denatured or degraded. We found that treatment with AMI-1 reduced tumor weight by 65.1 % compared with control-treated animals (Fig. 3b).

### AMI-1 inhibits PRMT5 activity and induces apoptosis in HCC cells

To further explore the mechanism of PRMT5 action in HCC, Western blot analysis was performed to determine protein levels of Bax and Bcl-2 in HCC cells. The results showed that AMI-1 increased Bax/Bcl-2 ratio associated with apoptosis relative to control cells (Fig. 3c). As shown in Fig. 3c, d, the expression of H4R3me2s protein was significantly decreased in AMI-1 treated cells compared with control cells. These results indicate that AMI-1 inhibits HCC growth, at least partially through inhibiting PRMT5 activity in HCC cells.

### PRMT5 inhibition promotes the apoptosis while inhibits the migration of HCC cells

**Fig. 4:**
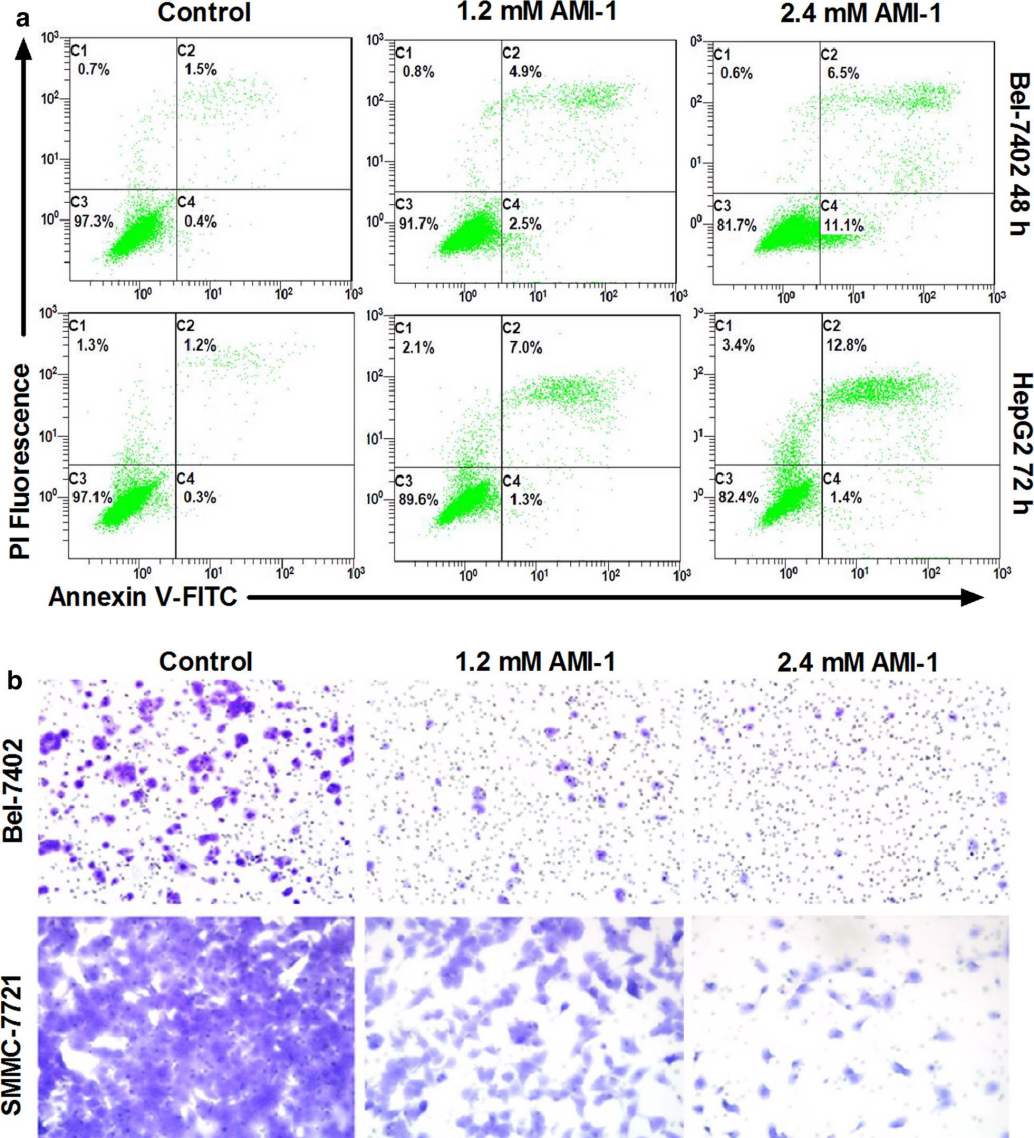
AMI-1 promotes the apoptosis and decreases migratory activity of HCC cells. **a** HCC cells were treated with vehicle or AMI-I and then stained by Annexin V-fluorescein isothicyanate (FITC) and propidium iodide (PI), followed by flow cytometry analysis. **b** AMI-1 decreased migratory activity of HCC cells measured by Transwell assay. Representative photos of stained cells are shown. Original magnification ×200. Control refers to vehicle-treated group

Inhibition of PRMT5 overexpression can induce apoptosis in different types of cancer [24, 31, 32]. To investigate the effect of AMI-1 on HCC cell viability, Bel-7402 and HepG2 cells were treated with either AMI-1 or vehicle only, and apoptosis was determined by Annexin V-FITC/propidium iodide staining and flow cytometry. As shown in Fig. 4a, PRMT5 inhibition by AMI-1 resulted in the induction of apoptosis/death in both cells compared with control cells. In addition, transwell assay showed that treatment of HCC cells with AMI-1 resulted in marked reduction in migration activity compared with control group (Fig. 4b). Furthermore, we employed siRNA to knockdown PRMT5 in HCC cells and found that PRMT5 siRNA increased the apoptosis while decreased the migration of HCC cells (Additional file 1: Figure 3).

## Discussion

PRMT5 emerges as an important regulator of multiple cellular processes and is expressed aberrantly in different types of cancer [11–15]. However, the role of PRMT5 in the pathogenesis of HCC and the relationship between PRMT5 expression and clinicopathological factors of HCC are largely unknown. In this study, we found that PRMT5 expression was markedly up-regulated in HCC tissues compared with NATs, and that higher expression of PRMT5 in the cytoplasm was significantly correlated with poor prognosis of HCC patients. Furthermore, we demonstrated that PRMT5 knockdown by siRNA could significantly inhibit the proliferation and colony formation of HCC cells, and repress cell cycle progression by inducing G0/G1 cell cycle arrest. Collectively, these data indicate that PRMT5 may function as an oncogene and is a key mediator in carcinogenesis and progression of HCC.

Wnt/β-catenin signaling is frequently activated in HCC, but the causes of its activation are not well understood [33]. Cytoplasmic and nuclear accumulations of β-catenin occur in 40–70 % of HCCs [34]. Upon Wnt activation, accumulated β-catenin enters the nucleus and binds to T-cell factor/lymphoid enhancer factor (TCF/LEF) to activate the transcription of Wnt target genes [35, 36]. A key event in both Wnt signaling and cancer cell proliferation is the regulation of β-catenin stability and activity. Our results showed that silencing PRMT5 significantly attenuates the expression levels of β-catenin and Cyclin D1. As Cyclin D1 is a downstream target of Wnt/β-catenin pathway and plays a key role as a regulator of cell proliferation [37], these data suggest that PRMT5 might mediate HCC tumor proliferation and development by regulating Cyclin D1 through Wnt/β-catenin pathway. This finding also suggests that PRMT5 mediated methylation is bi-functional, either repressing or promoting transcription, depending on what genes are expressed [38]. Additional studies will be required to distinguish the multiple potential mechanisms for PRMT5 function in different events of gene expression regulation.

There are several strategies to inhibit PRMT5 overexpression in cancer. In this study, we demonstrated that knockdown of PRMT5 by siRNA effectively inhibited HCC cell proliferation and colony formation in vitro, but treatment with siRNA is still in the experimental stage [39, 40]. Moreover, as no specific potent inhibitors are yet available for members of the PRMT enzyme family [25], our efforts focus on the discovery and development of small molecule inhibitors of PRMT5. AMI-1, a symmetrical sulfonated urea, was discovered by Cheng et al. [26]. However, AMI-1 has been applied to inhibit type I PRMT activity only in vitro experiments.

PRMTs are classified as either type I or type II. Both types can catalyze the formation of omega-N monomethylarginine (MMA) as an intermediate, and type I PRMTs (PRMT1, PRMT3, PRMT4, and PRMT6) lead to the production of asymmetrical dimethylarginine (aDMA), whereas type II PRMTs (PRMT5 and PRMT7) catalyze the formation of symmetrical dimethylarginine (sDMA) [41]. In this study, we first demonstrated that AMI-1 inhibited HCC growth in vitro and in vivo. Furthermore, we detected the expression of apoptosis related proteins such as Bax and Bcl-2 and found that AMI-1 up-regulated Bax/Bcl-2 ratio in HCC cells. AMI-1 also induced apoptosis and decreased migratory activity in several HCC cell lines. In addition, AMI-1 decreased the expression levels of H4R3me2s, a histone marker of PRMT5. These data suggest that AMI-1 exhibits anti-tumor effects against HCC, at least in part through inhibitting PRMT5.

## Conclusion

These results strongly indicate that PRMT5 is frequently up-regulated and inversely correlated with poor prognosis in HCC patients. PRMT5 might function as an oncogene partly by mediating the repression of β-catenin and its downstream target Cyclin D1 in HCC. Therefore, PRMT5 might be a potential biomarker and a promising therapeutic target for HCC.

## Additional file

10.1186/s12967-015-0721-8 **Figure 1.** Immunostaining of PRMT5 in normal and HCC tissue. In Negative control PRMT5 antibody was replaced with PBS. **Figure 2.** The endogenous expression level of PRMT5 in HCC cell lines and normal liver cell line. **Figure 3.** The effects of si-PRMT5 on the apoptosis and migration of HCC cells.
