# Supplementary material for: Targeting protein arginine methyltransferase 5 inhibits human hepatocellular carcinoma growth via the downregulation of beta-catenin
Source: J Transl Med. 2015 Nov 5;13:349. doi: 10.1186/s12967-015-0721-8 (PMC4635578; doi:10.1186/s12967-015-0721-8)
Supplement: Supplementary file 1 — 10.1186/s12967-015-0721-8 Figure 1. Immunostaining of PRMT5 in normal and HCC tissue. In Negative control PRMT5 antibody was replaced with PBS. Figure 2. The endogenous expression level of PRMT5 in HCC cell lines and normal liver cell line. Figure 3. The effects of si-PRMT5 on the apoptosis and migration of HCC cells. [file 12967_2015_721_MOESM1_ESM.doc]

**Targeting protein arginine methyltransferase 5 inhibits human hepatocellular carcinoma growth by decreasing beta-catenin**

**Supplementary Material**

**
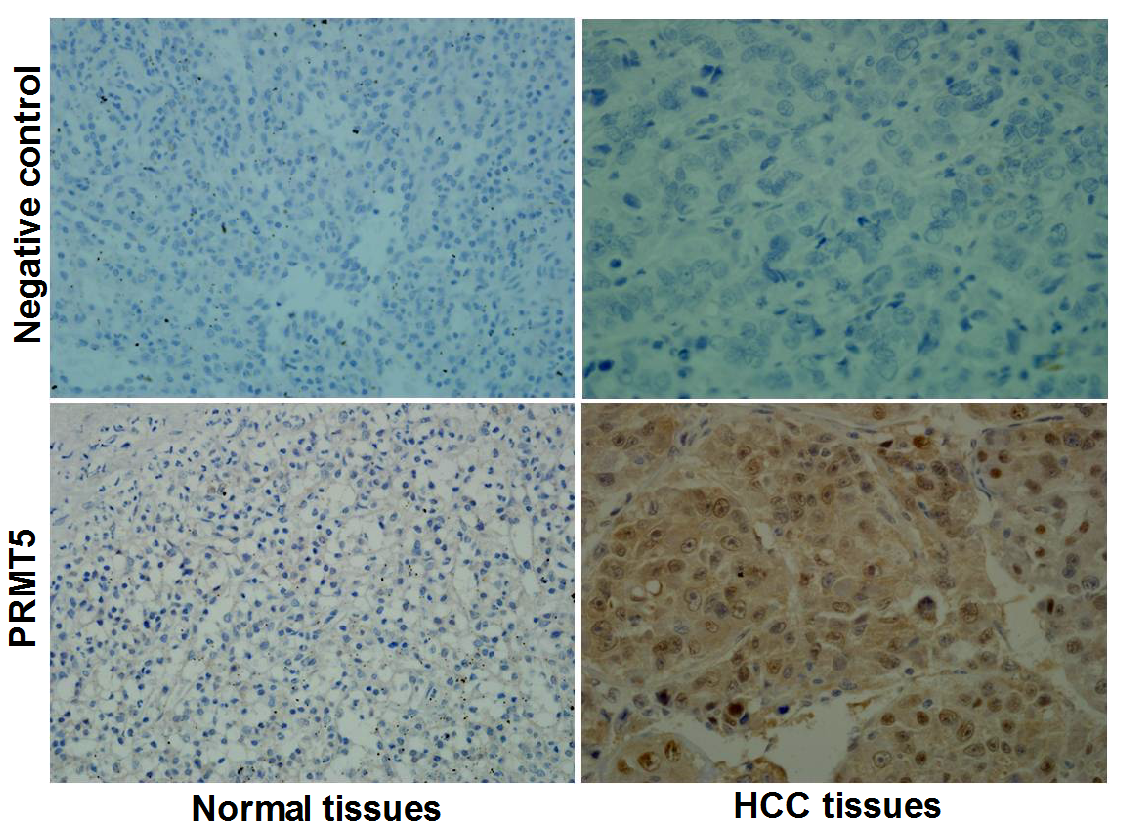
**

**Supplementary Figure 1: Immunostaining of PRMT5 in normal and HCC tissue.** In Negative control PRMT5 antibody was replaced with PBS.


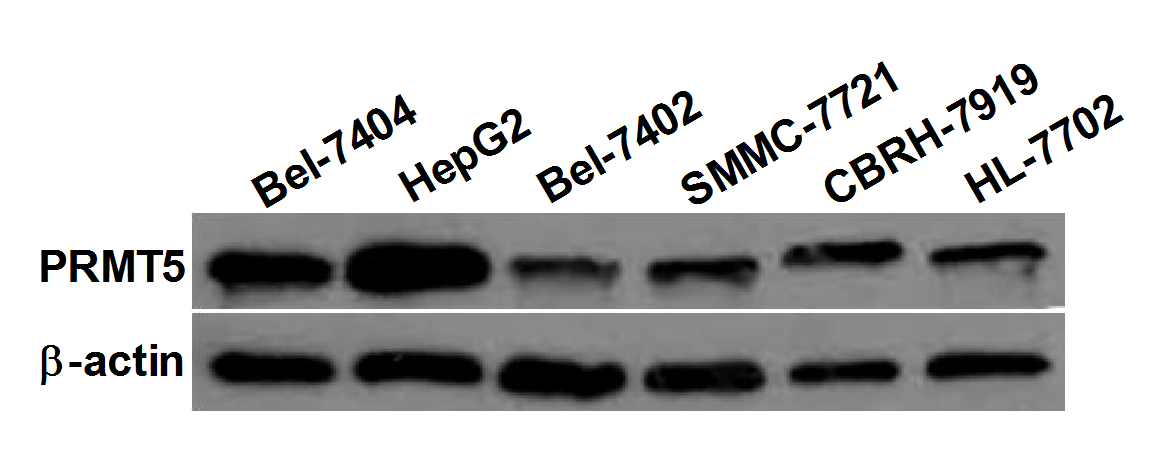


**Supplementary Figure 2: The endogenous expression level of PRMT5 in HCC cell lines and normal liver cell line**

**
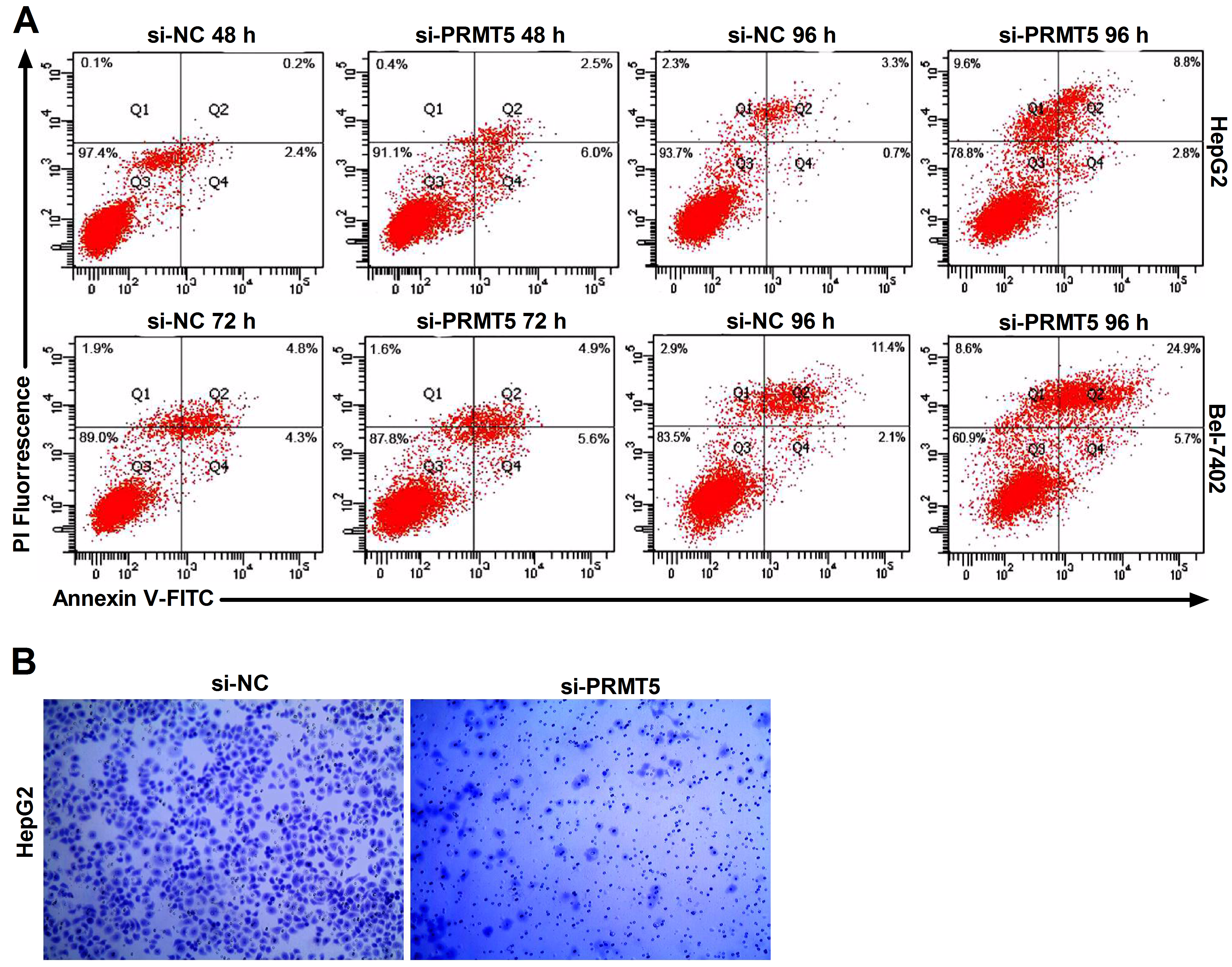
**

**Supplementary Figure 3: The effects of si-PRMT5 on the apoptosis and migration of HCC cells.** (**A)** HCC cells were treated with si-NC or si-PRMT5 and then stained by Annexin V-fluorescein isothicyanate (FITC) and propidium iodide (PI), followed by flow cytometry analysis. (**B)** si-PRMT5 decreased migratory activity of HCC cells measured by Transwell assay.
